# Pattern Distribution of Connexins in the Ortho- and Parakeratinized Epithelium of the Lingual Mucosa in Birds

**DOI:** 10.3390/cells12131776

**Published:** 2023-07-04

**Authors:** Kinga Skieresz-Szewczyk, Hanna Jackowiak

**Affiliations:** Department of Histology and Embryology, Faculty of Veterinary Medicine and Animal Science, Poznan University of Life Sciences, Wojska Polskiego 71C, 60-625 Poznan, Poland; hanna.jackowiak@up.poznan.pl

**Keywords:** connexins, lingual epithelia, cornification, birds

## Abstract

Connexins are important proteins involved in cell-to-cell communication and cytodifferentiation during renewal and cornification of the multilayered epithelia. So far, there is a lack of reports on this subject in birds’ structurally different ortho- and parakeratinized epithelium of the tongue. The study aims to describe the distribution and expression profiles of the α-connexins (Cx40 and 43) and β-connexins (Cx26, 30, and 31) in those epithelia in duck, goose, and domestic turkey. Research revealed the presence of the mentioned connexins and the occurrence of interspecies differences. Connexins form gap junctions in the cell membrane or are in the cytoplasm of keratinocytes. Differences in connexin expression were noted between the basal and intermediate layers, which may determine the proliferation of keratinocytes. Cx40, 43, and Cx30 in the gap junction of the keratinocytes of the intermediate layer are related to the synchronization of the cornification process. Because of the exfoliation of cornified plaques, a lack of connexins was observed in the cornified layer of orthokeratinized epithelium. However, in parakeratinized epithelium, connexins were present in the cell membrane of keratinocytes and thus maintained cellular integrity in gradually desquamating cells. The current studies will be useful in further comparative analyses of normal and pathological epithelia of the oral cavity in birds.

## 1. Introduction

Intercellular gap junctions in multilayered epithelia are responsible for the accurate processes of growth, proliferation, migration, and differentiation of keratinocytes and the maintenance of epithelial homeostasis [1,2,3,4]. They are functionally related to the process of keratinization or cornification [1,2,4]. Gap junctions allow for transport ions (i.e., Ca^2+^), metabolites, and other important signaling molecules of up to 1 kDa, i.e., cyclic adenosine monophosphate (cAMP), inositol trisphosphate (IP3), and diacylglycerol (DAG) [5,6,7,8,9,10]. 

Gap junctions are formed by two hemichannels—connexons. Each connexon is a hexamer composed of a connexin protein (Cx), whose polypeptide chain crosses the cell membrane four times [5,11]. Thus, the connexin has two extracellular loops and one intracellular loop. The connexin polypeptide chain’s N- and C-ends are located on the cytoplasmic side of the cell membrane. A disulfide bond connects the two extracellular loops to form a structure that allows the recognition of a connexin located within the opposite cell membrane. The connexins of neighboring cells contact each other, creating a gap junction 0.8–1.6 nm in diameter, whereas the neighboring cells’ membranes are about 2–4 nm apart [12,13,14].

The connexins that build the gap junction are formed in the rough endoplasmic reticulum, where they are folded and the oligomerization of connexin molecules into hemichannels starts [15]. The folded connexins then move into the cis-Golgi network, from where they are transported mainly along microtubules directly into the plasma membrane, where the hemichannels of adjacent cells join into gap junctions [6,12,16,17]. However, oligomerization of connexins can occur after leaving the ER in the trans-Golgi network (TGN) [17,18]. Due to this short half-life of connexins, the need for continuous replacement of connexin proteins results in free proteins in the cell cytoplasm in addition to the membrane-bound fraction [6,19,20].

The connexin gene family includes 20 genes in mice and 21 genes in humans, of which as many as 19 genes form specific or common ortholog pairs across vertebrate species [21]. The connexins are classified into subgroups based on amino acid and protein sequence: α-connexins (Cx33, 37, 40, 43, 46, 50, 59, and 62), β-connexins (Cx25, 26, 30, 31, and 32), and γ-connexins (Cx45 and 47) [21,22,23]. 

Connexins in multilayered epithelia have been extensively studied in the epidermis and occasionally in the oral cavity epithelia of the mouse, rat, and human [1,24,25,26,27,28,29,30]. These studies determined the presence of α-connexins (Cx40 and 43) and β-connexins (Cx26, 30, and 31). In avian species, connexins Cx26, 30, 31, 32, 43, and 45 were also analyzed during feather development. The results indicated that mesenchymal cell migration during feather elongation is complex and depends on calcium channels and connexin-43-based gap junctions [31,32].

There is a lack of reports about the occurrence of connexins in the species-specific multilayered epithelia of the mucosa of the beak cavity of birds, including the tongue. On the avian tongue, there are two types of cornified epithelia, i.e., ortho- and parakeratinized epithelium, composed only of three layers: basal, intermediate, and superficial [33,34,35,36,37,38,39]. These epithelia are found in functionally different areas of the avian lingual mucosa. The orthokeratinized epithelium is present in areas subject to mechanical stress during food intake, i.e., the lingual nail on the ventral surface of the lingual apex and on the conical papillae of the tongue. In contrast, the parakeratinized epithelium covers areas involved in food transport on the dorsal surface of the tongue [36,37,39].

Immunohistochemical and molecular studies of the cornification process of ortho- and parakeratinized epithelia by Skieresz-Szewczyk et al. [40,41,42] determined that there is a characteristic pattern of bird-specific alpha- and beta-keratins in the epithelia. Alpha-keratin is mainly accumulated in the lower layers of these epithelia, i.e., in the basal and intermediate layers, and its percentage is, on average, 38–45% [40,41]. Beta-keratin is present primarily in the cornified layer, whereby the orthokeratinized epithelium is characterized by a higher percentage of this keratin at 70% than the parakeratinized epithelium, in which the percentage of beta-keratin is about 61% [40,41]. Keratin-associated proteins (KAPs) (filaggrin and loricrin) and transglutaminase 1 (TGM-1) reveal stronger expression in the orthokeratinized epithelium than in the parakeratinized epithelium, which determines the formation of a more effective protective mechanical barrier [42]. The differences found in the expression of proteins in the cornification process are reflected in the diverse functions of these epithelia during food intake and transport into the oesophagus [40,41,42]. Extensive studies of the epidermis and skin appendages in mammals and birds have determined that transglutaminase is a conserved protein contributing to cornification [43]. It has also been determined that in mammals, birds, and reptiles, S100 fused-type proteins (SFTPs) involved in cornification, specifically cornulin and scaffoldin, share a common evolutionary origin [44]. At the same time, filaggrin is typical only in mammals [45].

The current study intends to verify whether (i) the α-connexins and β-connexins characterized previously in the epidermis and multilayered epithelia of the oral cavity in mammals are present in the ortho- and parakeratinized epithelium of the avian tongue and (ii) the expression profile of the examined connexins is different between ortho- and parakeratinized epithelium.

To address these two hypotheses, we did immunohistochemical analyses (IHC) on the distribution of the α-connexins Cx40 and 43 and β-connexins Cx26, 30, and 31 in the ortho- and parakeratinized epithelium of the tongue of duck, goose, and domestic turkey, representing different feeding groups.

The results will provide a better understanding of the synchronous cornification process of the ortho- and parakeratinized epithelium as well as the processes of their growth and renewal. Based on the present research, further discussion on the cornification of ectoderm-derived epithelia can be undertaken.

## 2. Materials and Methods

The research was conducted on the tongues of adult duck, goose, and domestic turkeys collected after slaughter from local breeders. To proceed with IHC analysis, three tongues were collected for each species, rinsed in saline, and fixed in 4% buffered formalin. The orthokeratinized epithelium from the ventral surface of the lingual apex and the parakeratinized epithelium from the dorsal surface of the lingual body were collected. Fixed tissue samples were subjected to dehydration in increasing ethanol concentrations (70–96%) and routinely embedded in Paraplast^®^ (Sigma-Aldrich, Taufkirchen, Germany). Paraplast blocks were cut into serial sections of 4.5–5 um. IHC analysis was performed according to the protocol by Skieresz-Szewczyk et al. [40]. Tissue samples were deparaffinized for 12 h at 60 °C and then washed in three changes of xylene and in a series of decreasing alcohol concentrations (99.8–80%). Exposure of antigenic determinants was performed using the buffer DakoTarget Retrieval Solution, pH 5.9 (Agilent, Santa Clara, CA, USA) in a water bath (type WB-4 MS, Biosan, Warsaw, Poland) at 97 °C for 40 min, and then tissue samples were cooled for 20 min at room temperature and washed in double-distilled water. A 3% hydrogen peroxide solution was used to block non-specific immunoglobulin binding sites, in which tissue samples were immersed at room temperature for 10 min. After that, the tissue samples were washed in two changes of double-distilled water and phosphate-buffered saline (PBS, pH 7.4) for 10 min. Tissue samples were incubated with the primary antibody at 37 °C in a humid chamber for 30 min. The following antibodies were used: anti-Cx26 (dilution 1:100, cat. MABT198, Merck, Poznan, Poland); anti-Cx30 (dilution 1:200, cat. 71-2200, TermoFisher Scientific, Warsaw, Poland anti-Cx31 (dilution 1:100, cat. 36-5100, Invitrogen); anti-Cx40 (dilution 1:50, cat.36-4900, TermoFisher Scientific, Warsaw, Poland); and anti-Cx43 (dilution 1:100, cat.71-0700, TermoFisher Scientific, Warsaw, Poland). After incubation, histological sections were rinsed in PBS (pH 7.4) for 10 min and incubated in a humid chamber for 20 min at 37 °C with goat anti-rabbit/mouse IgG (Polymer Labeled HRP-Anti-Rabbit/Mouse; Agilent, Santa Clara, CA, USA). Afterwards, tissue samples were washed in PBS (pH 7.4) for 10 min. To visualize the binding of the secondary antibodies, the substrate—DAB+ chromogen solution (Agilent, Santa Clara, CA, USA)—was used. Tissue samples were counterstained in Mayer’s hematoxylin for 5 min and dehydrated in a series of increasing alcohol concentrations (80–99.8%) and acetone. The first antibody was omitted for negative controls, and the tissue samples were incubated with PBS (pH 7.4) (Figures S1–S5 in the Supplementary Materials). Microscopic observation of the IHC analyses of the examined connexins was performed on five samples using an Axioscope 2plus light microscope (Zeiss, Oberkochen, Germany). All images in the manuscript are documented using the lens 63×/1.4 oil immersion (Plan-APOCHROMAT, Zeiss). Images in the supplementary materials, i.e., the orthokeratinized epithelium and parakeratinized epithelium in the duck are documented by lens 20×/0.50 (Plan-NEOFLUAR, Zeiss) and the parakeratinized epithelium in the goose and turkey by lens 5×/0.15 (Plan-NEOFLUAR, Zeiss). According to Polish law and the EU directive no. 2010/63/EU, the present study does not require approval of the Local Ethical Committee for Experiments on Animals in Poznan.

## 3. Results

### 3.1. Immunohistochemical Analysis

The results of immunohistochemical analyses with the anti-Cx26, 30, 31, 40, and 43 antibodies are included in Table 1 and Table 2.

#### 3.1.1. Cx26 Analysis

**Figure 1:**
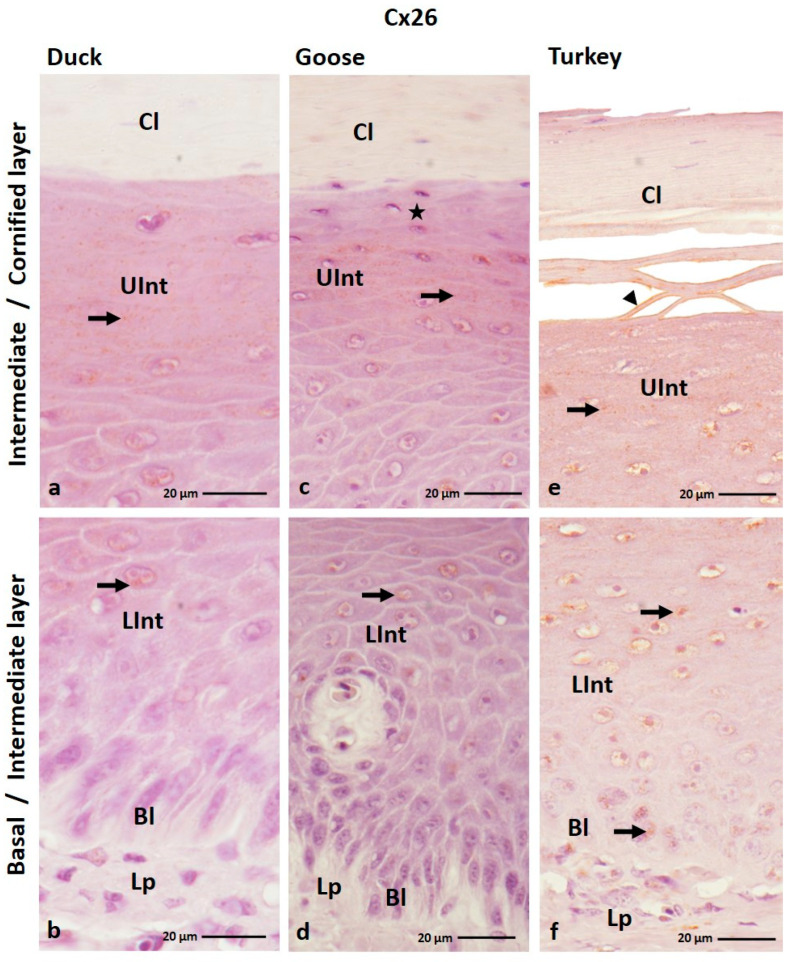
(**a**–**f**) The cross-section of the orthokeratinized epithelium. IHC staining of the Cx26. Bl—basal layer; Lp—lamina propria of the mucosa; LInt—the lower part of the intermediate layer; UInt—upper part of the intermediate layer; Cl—cornified layer. Arrows indicate the positive color reactions in the cell cytoplasm. The arrowhead shows the cell membrane with a positive color reaction. The asterisk marks the cells with no positive color reactions.

**Figure 2:**
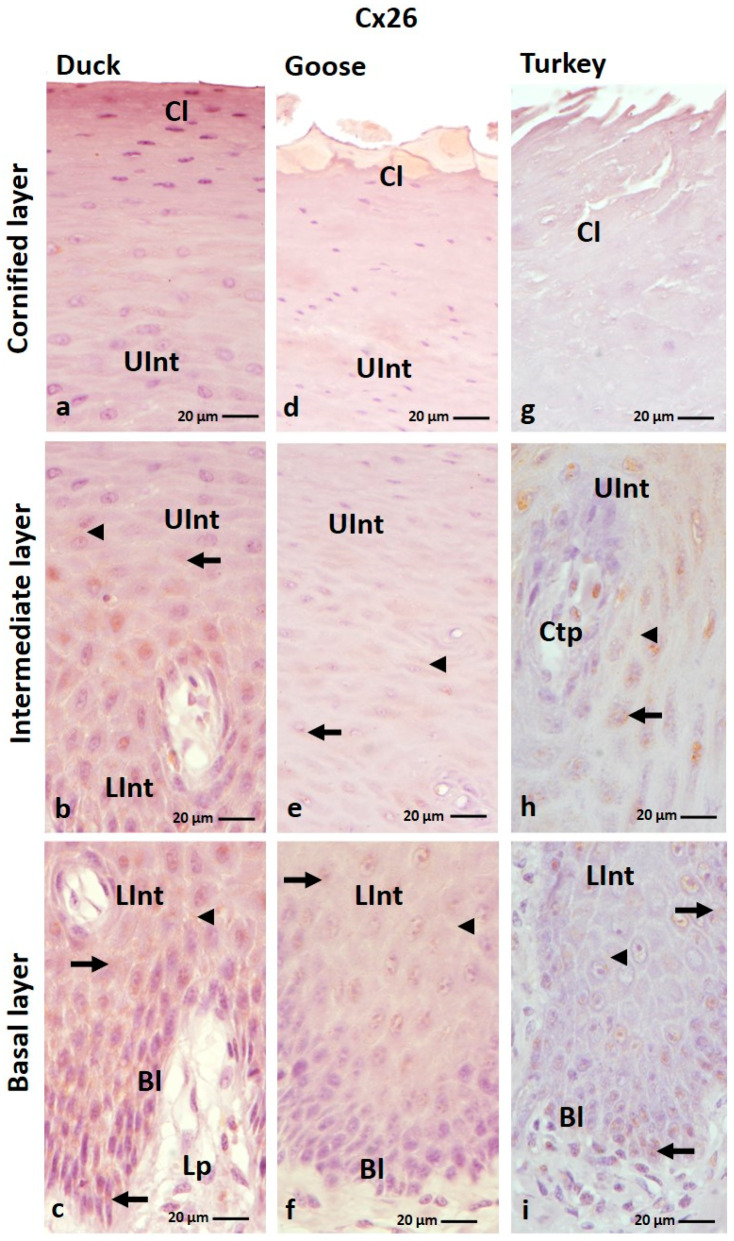
(**a**–**i**) The cross-section of the parakeratinized epithelium. IHC staining of the Cx26. Bl—basal layer; Lp—lamina propria of the mucosa; LInt—the lower part of the intermediate layer; UInt—upper part of the intermediate layer; Cl—cornified layer; Ctp—connective tissue papillae. Arrows point to the positive color reaction in the cell cytoplasm. The arrowheads show the cell membranes with a positive color reaction.

The result of IHC staining showed that connexin 26 is present mainly in the intermediate layer of the ortho- and parakeratinized epithelium of the tongue of the studied bird species (Figure 1a–f and Figure 2a–i; Table 1). Positive color reactions were also observed in the basal layer of the ortho- and parakeratinized epithelium, in the cornified layer of the orthokeratinized epithelium in the domestic turkey, and in the basal layer of the parakeratinized epithelium in the domestic duck (Figure 1e,f and Figure 2c,i).

In the orthokeratinized epithelium, Cx26 is mainly found in the cell cytoplasm (Figure 1a–f; Table 1). The exception is the cornified layer in domestic turkey, where a weak color reaction in 7–8 cell layers was recorded in the cell membrane (Figure 1e, Table 1). In the lower part of the intermediate layer, weak color reactions were observed in single cells around the cell nucleus in domestic goose and duck and all cells in domestic turkey (Figure 1b,d,f; Table 1). In the upper part of the intermediate layer, weak color reactions were recorded throughout the cytoplasm of all cells in duck and domestic turkey (Figure 1a,e; Table 1). The exception is the domestic goose, where 3–4 layers of cells of the upper part of the intermediate layer, arranged just below the cornified layer of the orthokeratinized epithelium, show no color reactions (Figure 1c; Table 1). 

In the parakeratinized epithelium, positive color reactions with the anti-Cx26 antibody in the basal layer were observed in the cell membrane and cell cytoplasm in the domestic duck and only in the cell cytoplasm in the domestic turkey (Figure 2c,i; Table 1). The color responses were medium and weak, respectively (Table 1). In the lower and upper parts of the intermediate layer, Cx26 is present in the cell cytoplasm and the cell membrane in all investigated bird species, and the level of color response is generally weak (Figure 2a–i; Table 1). The exception is the lower part of the intermediate layer in the domestic duck, where a medium color response was recorded (Figure 2c; Table 1). Attention should also be paid to the intermediate layer in the domestic turkey, where positive color reactions in both parts are present in the areas between the connective tissue papillae (Figure 2h; Figure S1).

#### 3.1.2. Cx30 Analysis

**Figure 3:**
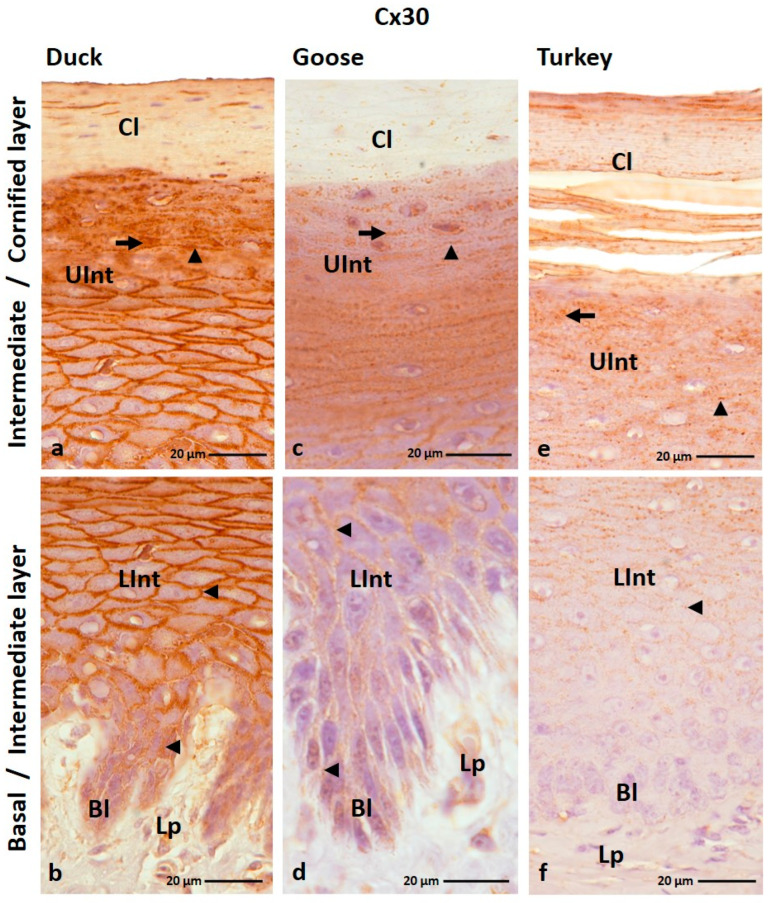
(**a**–**f**) The cross-section of the orthokeratinized epithelium. IHC staining of the Cx30. Bl—basal layer; Lp—lamina propria of the mucosa; LInt—the lower part of the intermediate layer; UInt—upper part of the intermediate layer; Cl—cornified layer. Arrows point to the positive color reaction in the cell cytoplasm. The arrowheads show a positive color reaction in the cell membranes.

**Figure 4:**
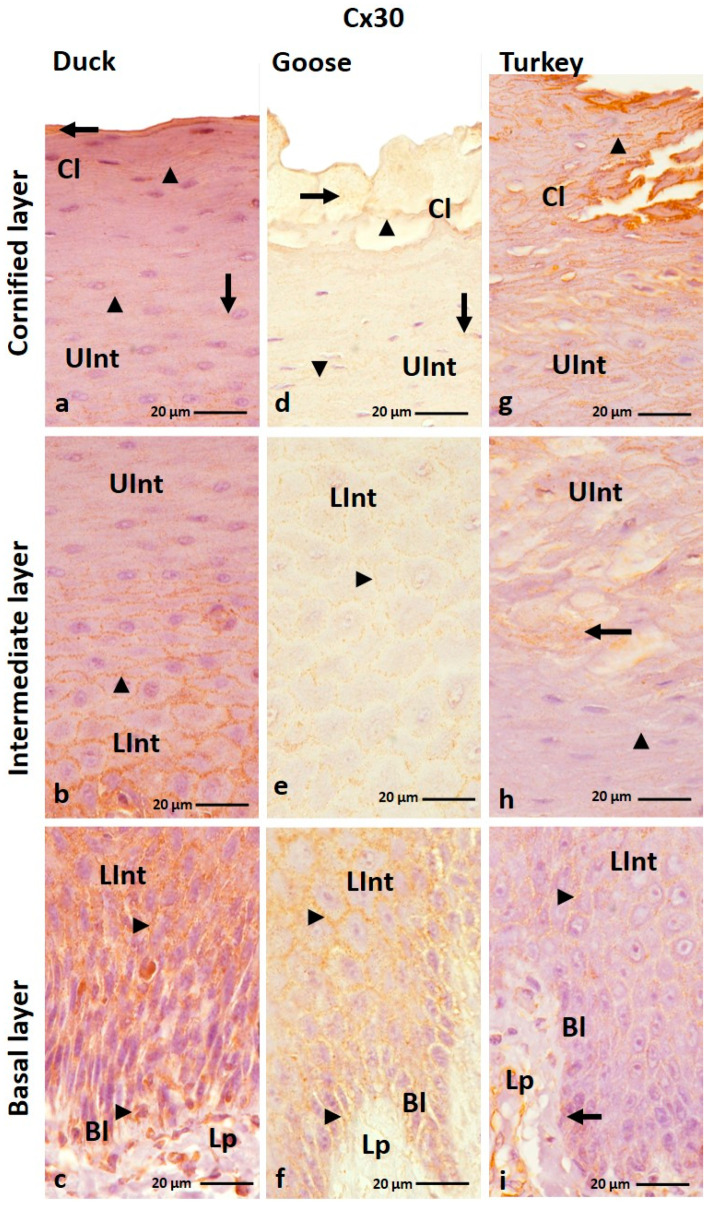
(**a**–**i**) The cross-section of the parakeratinized epithelium. IHC staining of the Cx30. Bl—basal layer; Lp—lamina propria of the mucosa; LInt—the lower part of the intermediate layer; UInt—upper part of the intermediate layer; Cl—cornified layer. Arrows show the positive color reaction in the cell cytoplasm. The arrowheads point to the cell membranes with a positive color reaction.

The IHC reaction with anti-Cx30 antibody revealed the presence of Cx30 in all layers of the ortho- and parakeratinized epithelium except the basal layer of the orthokeratinized epithelium in the domestic turkey (Figure 3a–f and Figure 4a–i; Table 1).

In the orthokeratinized epithelium, Cx30 is mostly present in the cell membrane of cells of the basal layer and the lower part of the intermediate layer in all studied bird species, where medium and strong color responses were recorded, respectively (Figure 3b,d,f; Table 1). Whereby in the domestic turkey, the color response in the lower part of the intermediate layer is medium (Table 1). In the upper part of the intermediate layer of the orthokeratinized epithelium, Cx30 is present not only in the cell membrane but also in the cell cytoplasm in both goose, duck, and domestic turkey (Figure 3a,c,e; Table 1). A medium color response is in the domestic turkey, and a strong one is in the domestic duck (Table 1). In the domestic goose, a strong color response was observed in the lower portion of the upper part of the intermediate layer and a medium color response in the 4–5 cell layers just below the cornified layer (Figure 3c; Table 2). In the cornified layer, generally, no positive color reaction was recorded. Only in a few cell layers just above the upper part of the intermediate layer were there weak color reactions in the cell cytoplasm (Figure 3a,c,e; Table 1). There were 3–4 cell layers in the domestic duck, and in the domestic goose and turkey, there were about 6–7 cell layers. 

The parakeratinized epithelium was generally characterized by a medium color response in the cell membrane of the cell in the basal layer and the lower part of the intermediate layer in the examined bird species (Figure 4b,c,f,i; Table 1). The exception is the basal layer in the domestic turkey, where color reactions at a weak level were recorded only in the cell cytoplasm around the cell nuclei (Figure 4i; Table 1). Interestingly, in goose and domestic duck, the level of color reaction in the lower part of the intermediate layer decreases towards the surface of the parakeratinized epithelium (Figure 4b,c,e,f). In the upper part of the intermediate layer, weak color reactions were observed in the cell membrane and cell cytoplasm in goose and domestic duck and only in the cell membrane in domestic turkey (Figure 4a,b,d,h; Table 2). Only the 3–4 cell layers of the upper part of the intermediate layer located just below the cornified layer in the domestic turkey showed positive color reactions in the cell cytoplasm (Figure 4h). Positive color reactions in both parts of the intermediate layer in the domestic turkey are present between the connective tissue papillae (Figure S2). In the cornified layer of the parakeratinized epithelium, Cx30 is present in both the cell membrane and cytoplasm in goose and domestic duck, where a weak color reaction was observed (Figure 4a,d; Table 1). In domestic turkey, Cx30 is present only in the cell membrane of cells of the cornified layer, and the level of color reaction is medium (Figure 4g; Table 1).

#### 3.1.3. Cx31 Analysis

**Figure 5:**
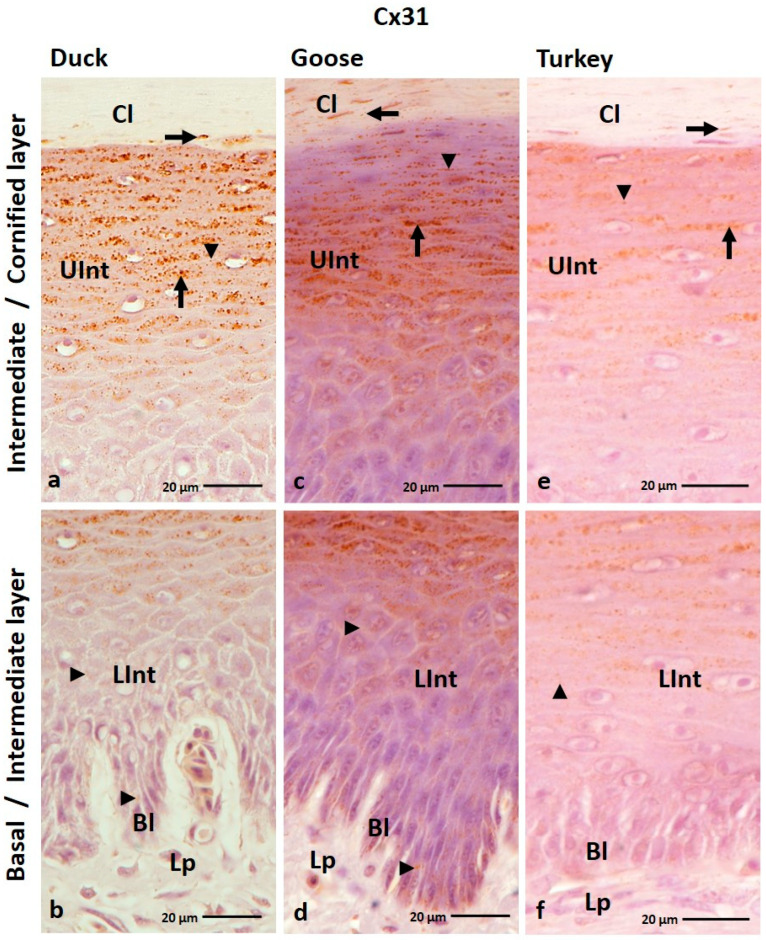
(**a**–**f**) The cross-section of the orthokeratinized epithelium. IHC staining of the Cx31. Bl—basal layer; Lp—lamina propria of the mucosa; LInt—the lower part of the intermediate layer; UInt–upper part of the intermediate layer; Cl—cornified layer. Arrows point to the positive color reaction in the cell cytoplasm. The arrowheads show a positive color reaction in the cell membranes.

**Figure 6:**
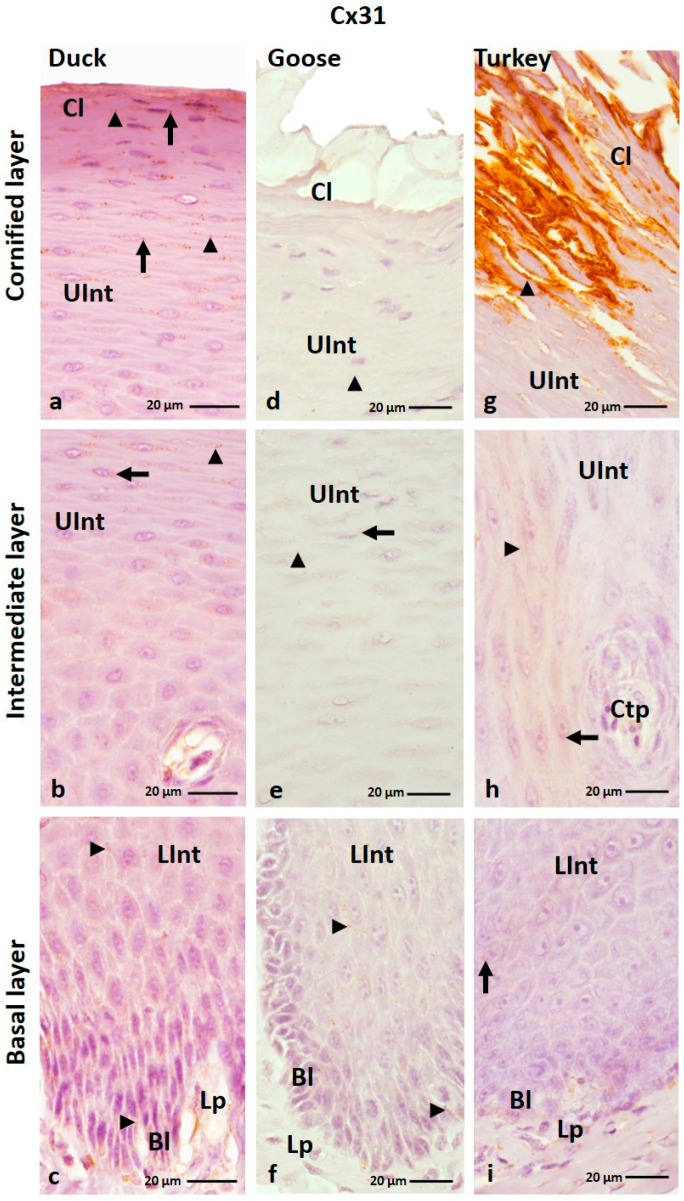
(**a**–**i**) The cross-section of the parakeratinized epithelium. IHC staining of the Cx31. Bl—basal layer; Lp—lamina propria of the mucosa; LInt—the lower part of the intermediate layer; UInt—upper part of the intermediate layer; Cl—cornified layer. Arrows point to the positive color reaction in the cell cytoplasm. The arrowheads show a positive color reaction in the cell membranes.

IHC analysis of the Cx31 antibody showed that it is generally present in all layers of the ortho- and parakeratinized epithelium in the three examined bird species (Figure 5a–f and Figure 6a–i; Table 1). The exceptions are the basal layer in the parakeratinized epithelium in the domestic turkey and the cornified layer in the parakeratinized epithelium in the domestic goose (Figure 6d,i; Table 1).

In the orthokeratinized epithelium, the basal layer and lower part of the intermediate layer showed a weak color reaction with Cx31 only in the cell membrane in all studied bird species (Figure 5b,d,f; Table 1). In the upper part of the intermediate layer, a medium color reaction was observed in both the cell membrane and the cell cytoplasm in duck, goose, and domestic turkey (Figure 5a,c,e; Table 1). In general, the cornified layer of the orthokeratinized epithelium does not show a positive color reaction with the anti-Cx31 antibody. The exception is in the 2–3 layers of cells located just above the upper part of the intermediate layer, where weak color reactions were noted in the cell cytoplasm (Figure 5a,c,e; Table 1). 

In the parakeratinized epithelium, Cx31 showed generally weak color reactions in the epithelium in all bird species (Table 1). Color reactions in the basal layer and lower part of the intermediate layer were recorded in the cell membrane in goose and duck and in the cell membrane and cell cytoplasm in turkey (Figure 6c,f,i; Table 1). In the upper part of the intermediate layer and the cornified layer, positive color reactions are present in the cell membrane and cell cytoplasm (Figure 6a–h; Table 1). The exception is the domestic turkey, in which a positive medium color reaction in the cornified layer is only visible in the cell membrane (Figure 6g; Table 1). Attention should also be drawn to the lower and upper parts of the intermediate layer of the parakeratinized epithelium in the domestic turkey, where only part of the cells in the areas between the connective tissue papillae show positive color reactions with the anti-Cx31 antibody (Figure 6h,i; Figure S3). In addition, in the domestic turkey, only part of the cells in the upper part of the intermediate layer just below the cornified layer show a weak color reaction (Figure S3).

#### 3.1.4. Cx40 Analysis

**Figure 7:**
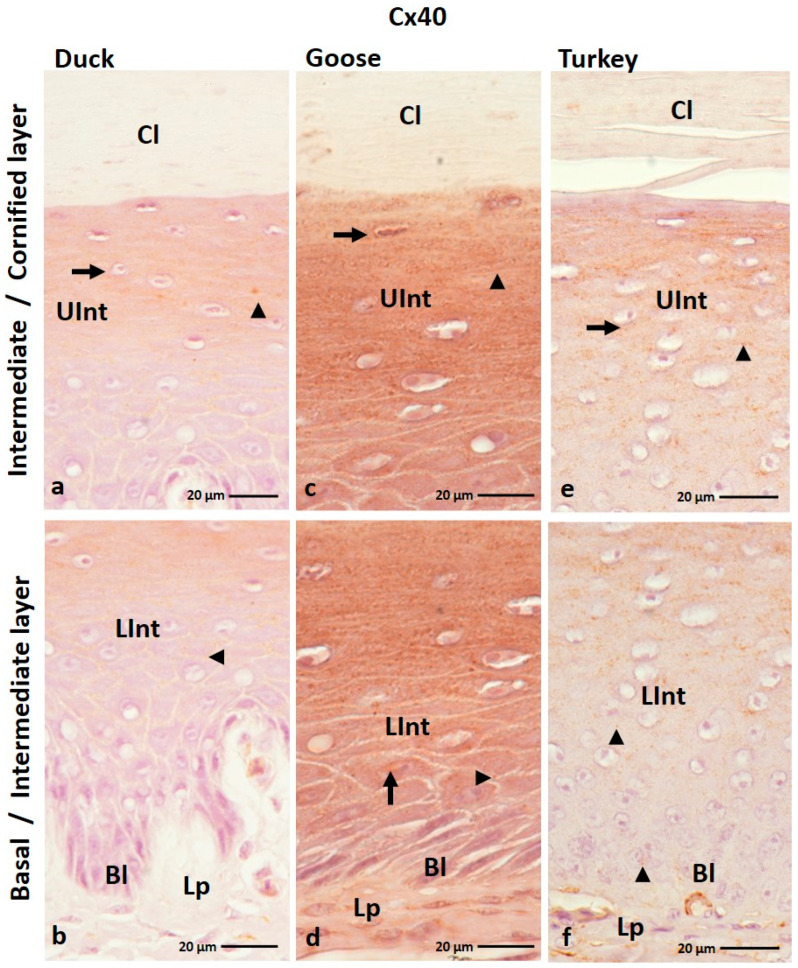
(**a**–**f**) The cross-section of the orthokeratinized epithelium. IHC staining of the Cx40. Bl—basal layer; Lp—lamina propria of the mucosa; LInt—the lower part of the intermediate layer; UInt—upper part of the intermediate layer; Cl—cornified layer. Arrows point to the positive color reaction in the cell cytoplasm. The arrowheads show the cell membranes with a positive color reaction.

**Figure 8:**
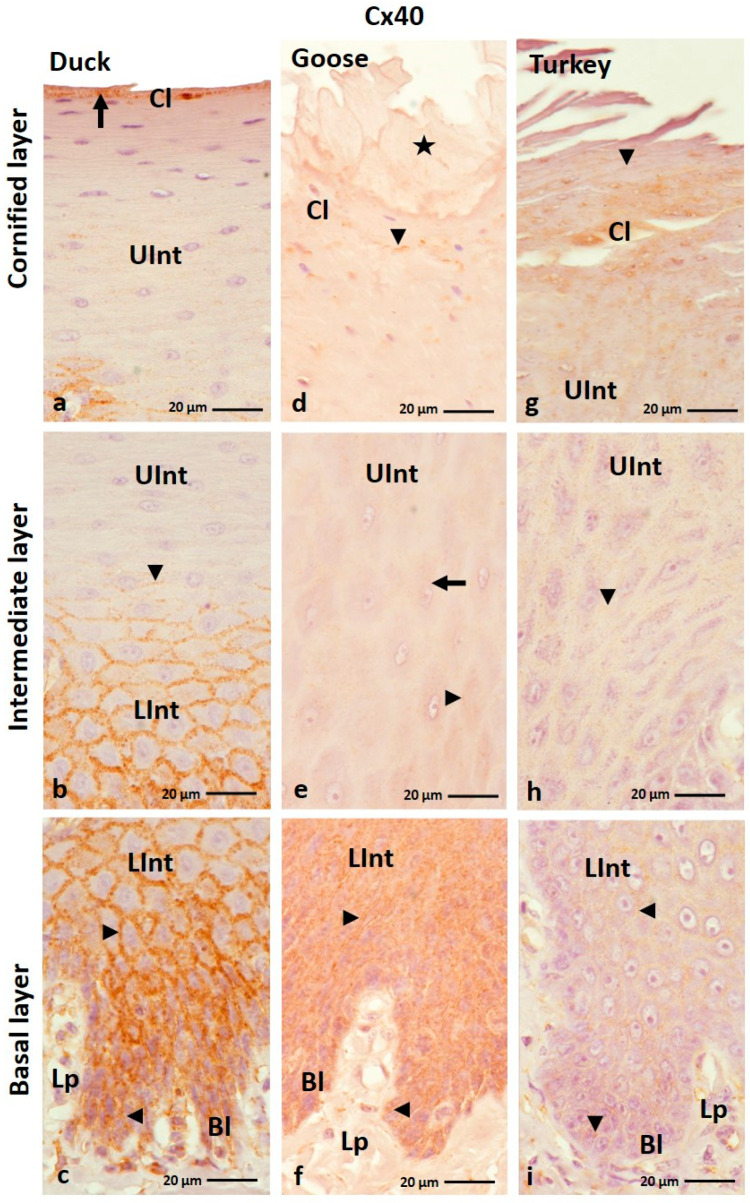
(**a**–**i**) the cross-section of the parakeratinized epithelium. IHC staining of the Cx40. Bl—basal layer; Lp—lamina propria of the mucosa; LInt—the lower part of the intermediate layer; UInt—upper part of the intermediate layer; Cl—cornified layer. Arrows point to the positive color reaction in the cell cytoplasm. The arrowheads show a positive color reaction in the cell membranes. Asterisk marks to the superficial cell of the cornified layer with no positive color reaction.

The results of IHC staining with an anti-Cx40 antibody showed that this protein is generally present in all layers of the ortho- and parakeratinized epithelium except the cornified layer of the orthokeratinized epithelium (Figure 7a–f and Figure 8a–i).

In the orthokeratinized epithelium, Cx40 is located in the cell membrane of the cells of the basal layer and lower part of the intermediate layer in all studied avian species, where the level of color response is weak (Figure 7b,d,f; Table 2). The exception is the lower part of the intermediate layer in the domestic goose, where a color reaction at a medium level was observed both in the cell membrane and cell cytoplasm (Figure 7d; Table 2). In the upper part of the intermediate layer, medium color reactions with Cx40 are seen in the cell membrane and in the cell cytoplasm (Figure 7a,c,e; Table 2). 

In the parakeratinized epithelium, positive color reactions with Cx40 were shown in the cell membrane of the basal layer and the lower part of the intermediate layer in all examined bird species (Figure 8c,f,i). Strong color reactions were observed in duck and domestic goose, and medium color reactions were observed in domestic turkey (Table 2). In the upper part of the intermediate layer, weak color reactions in duck and domestic turkey were recorded in the cell membrane (Figure 8a,b,h; Table 2). Whereby cells of the upper part of the intermediate layer in domestic turkey, located just below the cornified layer, show color reactions also in the cell cytoplasm (Figure 8g). In the domestic goose, weak color reactions in the upper part of the intermediate layer were observed in the cell membrane and in the cell cytoplasm (Figure 8e; Table 2). Interestingly, in the intermediate layer of the domestic turkey, positive color reactions were present in the areas between the connective tissue papillae (Figure S4). The cornified layer of the parakeratinized epithelium in the studied bird species was characterized by a weak color reaction in the cell cytoplasm in the domestic duck and in the cell cytoplasm and cell membrane in the domestic goose and turkey (Figure 8a,d,g; Table 2). The superficial cells of the cornified layer of the parakeratinized epithelium in the domestic goose do not show a positive color response (Figure 8d).

#### 3.1.5. Cx43 Analysis

**Figure 9:**
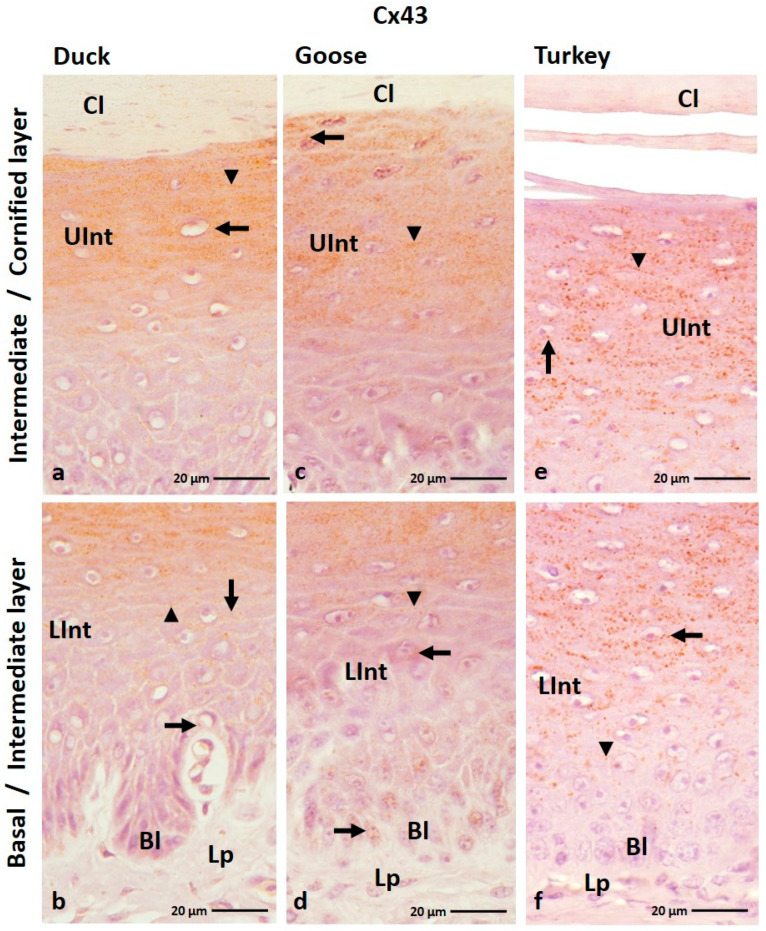
(**a**–**f**) The cross-section of the orthokeratinized epithelium. IHC staining of the Cx43. Bl—basal layer; Lp—lamina propria of the mucosa; LInt—the lower part of the intermediate layer; UInt—upper part of the intermediate layer; Cl—cornified layer. Arrows point to the cells with the positive color reaction in the cell cytoplasm. The arrowheads show the cell membranes with a positive color reaction.

**Figure 10:**
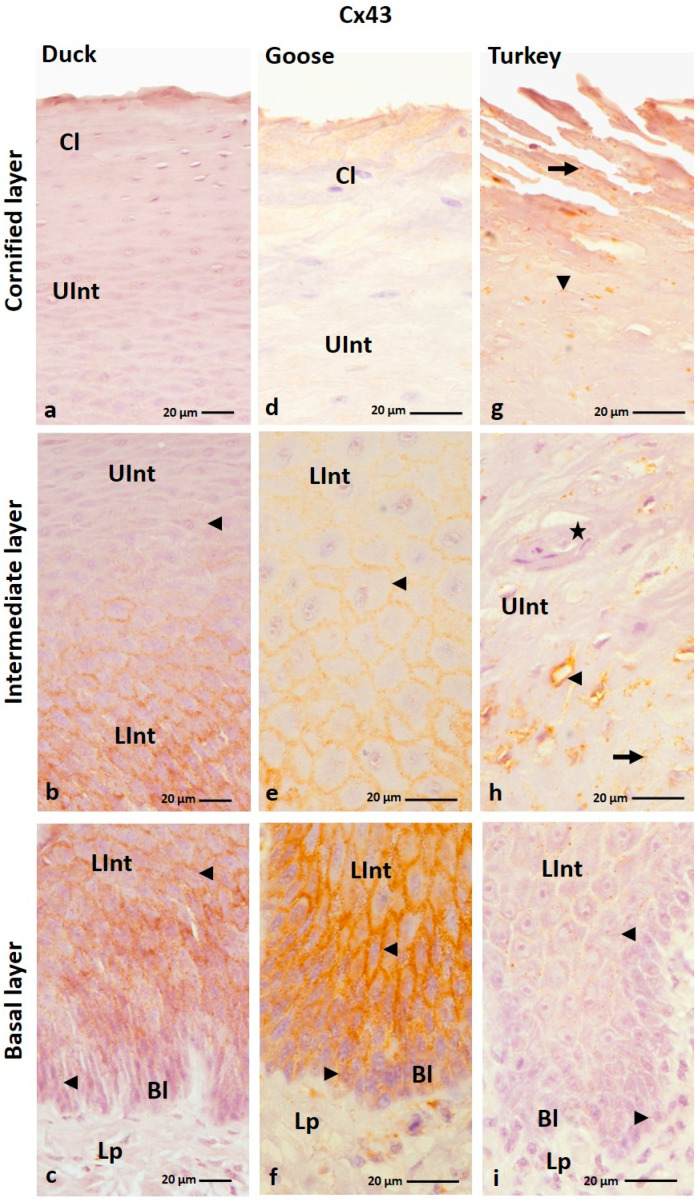
(**a**–**i**) The cross-section of the parakeratinized epithelium. IHC staining of the Cx43. Bl—basal layer; Lp—lamina propria of the mucosa; LInt—the lower part of the intermediate layer; UInt—upper part of the intermediate layer; Cl—cornified layer. Arrows show the positive color reaction in the cell cytoplasm. The arrowheads point to the cell membranes with a positive color reaction. The asterisk marks the area where only some cells give a positive color reaction.

The IHC reaction with anti-Cx43 antibody showed that Cx43 is present in all layers of the parakeratinized epithelium, except the cornified layer in the examined bird species and the basal layer in the domestic turkey (Figure 9a–f; Table 2). In the parakeratinized epithelium, Cx43 is mainly present in the basal and intermediate layers in goose, duck, and domestic turkey and in the cornified layer in domestic turkey (Figure 10a–i; Table 2).

In the domestic goose and duck, the basal layer of the orthokeratinized epithelium is characterized by a weak color reaction in the cell cytoplasm around the cell nuclei (Figure 9b,d; Table 2). In the lower part of the intermediate layer, positive color reactions were observed in the cell cytoplasm and the cell membrane (Figure 9b,d,f). At the same time, in the lower part of the intermediate layer, color reactions were weak in the domestic goose and duck and medium in the domestic turkey (Table 2). In the upper part of the intermediate layer, generally strong color responses were observed in the cell cytoplasm and cell membrane in all studied bird species (Figure 9a,c,e; Table 2).

In the parakeratinized epithelium, the basal layer shows weak color reactions in the cell membrane in duck and domestic turkey and strong reactions in domestic goose (Figure 10c,f,i; Table 2). In the intermediate layer, color reactions were observed mainly in the cell membrane (Figure 10b,c,e,f,i). The exception is the upper part of the intermediate layer in domestic turkey, where positive color reactions were weakly recorded in the cell membrane and cell cytoplasm (Figure 10h). Color reactions in the lower part of the intermediate layer are strong in the domestic goose and duck and medium in the domestic turkey (Table 2). Weak color reactions were observed in the upper part of the intermediate layer (Table 2). In the domestic turkey, only part of the cells in the intermediate layer, in the areas between the connective tissue papillae, show positive color reactions with the anti-Cx43 antibody (Figure S5). Interestingly, cells located just below the cornified layer of the parakeratinized epithelium in duck and goose did not show a positive color reaction with Cx43 (Figure 10a,d; Table 2). In the domestic turkey, the color reaction is still observed in some cells (Figure 10h). Only in the domestic turkey, a positive color reaction at a weak level was visible in the cell membrane and cell cytoplasm of the cells of the cornified layer of the parakeratinized epithelium (Figure 10g; Table 2).

## 4. Discussion

### 4.1. The Pattern of Connexin Distribution in the Ortho- and Parakeratinized Epithelium of the Tongue of the Studied Bird Species

As mentioned in the introduction, the distribution of α-connexins Cx40 and 43 and β-connexins Cx26, 30 and 31 has been studied in the epidermis in mouse, rat, and human [1,24,27,29,30], the epithelium in the buccal cavity in mouse and human, and the lingual epithelium in hamster [25,28,30]. There is a lack of reports on this research subject in birds.

**Figure 11:**
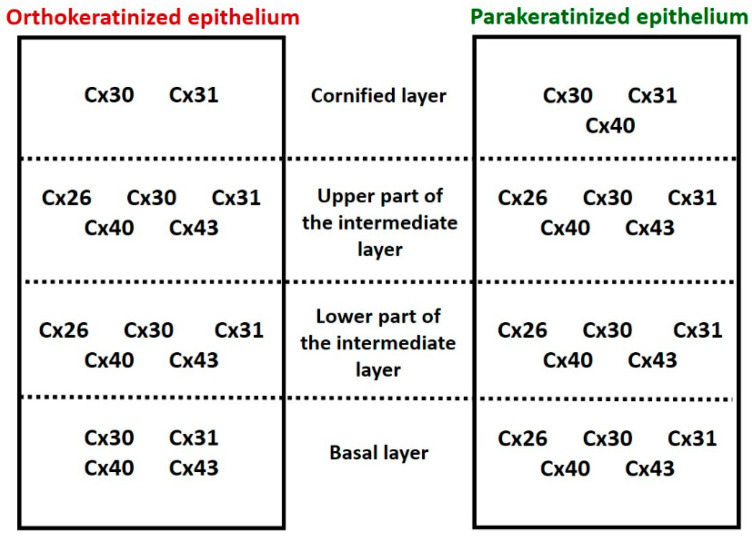
Distribution of the connexins in the ortho- and parakeratinized epithelium.

The present IHC analysis indicates, for the first time in birds, the presence of all the above-mentioned connexins in the two types of lingual cornified epithelia, i.e., in the ortho- and parakeratinized epithelium. We determine that α-connexins Cx40 and 43, as well as β-connexin Cx30, are generally characterized by medium-to-strong level of expression and are present in all layers of the ortho- and parakeratinized epithelium (Figure 11). The exceptions are (i) the cornified layer of the orthokeratinized epithelium, where Cx40 is absent, and (ii) the cornified layer of both types of cornified epithelia of the tongue, where Cx43 was not observed. The β-connexins Cx26 and 31 are characterized by a weak level of expression in both types of cornified epithelia, whereby Cx26 is mainly present in the intermediate layer and Cx31 is located in all epithelial layers.

The expression profile of the studied connexins in the ortho- and parakeratinized epithelium of the examined avian tongue indicates the presence of species-specific features. The highest number of species-specific features were observed in granivorous domestic turkeys. These features included (i) the absence of Cx43 in the basal layer of the orthokeratinized epithelium and its presence in the cornified layer of the parakeratinized epithelium in the domestic turkey, (ii) the presence of Cx26 in the basal layer of the parakeratinized epithelium in turkey and domestic duck and in the orthokeratinized epithelium in domestic turkey and in the cornified layer of the orthokeratinized epithelium in domestic turkey, (iii) the absence of Cx31 in the basal layer of the parakeratinized epithelium in domestic turkey and in the cornified layer of the parakeratinized epithelium in domestic goose (iv), the absence of Cx30 in the basal layer of the orthokeratinized epithelium in domestic turkey.

Comparative analysis showed that the expression level of α-connexin Cx40 in the basal layer and lower part of the intermediate layer of the orthokeratinized epithelium is weaker than in the parakeratinized epithelium. Differences were also observed in the expression of Cx30, 31, 40, and 43 in the intermediate layer of both types of lingual epithelia. Cx30 expression in the intermediate layer of the orthokeratinized epithelium is generally stronger than in the parakeratinized epithelium. At the same time, Cx30 expression in the lower and upper parts of the intermediate layer of the orthokeratinized epithelium does not change, while a decrease in expression in the upper part of the intermediate layer is present in the parakeratinized epithelium. In contrast, Cx31, 40 and 43 show a similar trend of changing expression levels, i.e., an increase in expression was observed in the upper part of the intermediate layer of the orthokeratinized epithelium and a decrease in expression was noted in the parakeratinized epithelium. The exception is Cx31, whose expression in the lower and upper parts of the intermediate layer of the parakeratinized epithelium does not change. We also noted the differences in expression in the cornified layer, where Cx30 and 31 show weaker expression in the orthokeratinized epithelium than in the parakeratinized epithelium. In general, only Cx26 does not show differences in expression level within the different layers of the two types of lingual cornified epithelia in birds. 

Only reports of connexin distribution in multilayered epithelia of the buccal cavity refer to the presence of Cx26, 30, and 43. Cx26 reveals generally strong expression in upper epithelial layers and in interphase cells of the basal layer in the epithelium of the buccal cavity in mice and humans [28,30] In the highly cornified epithelium of the hamster tongue, Cx26 is present mainly in the granular layer, and only single staining spots are observed in the upper part of the cornified layer [25]. We state that the expression profile of Cx26 in the ortho- and parakeratinized epithelium of the avian tongue is similar to that of the lingual epithelium in a hamster. In the case of Cx30, the ortho- and parakeratinized epithelium, as well as the epithelium of the buccal cavity in mice reveals strong expression in the uppermost layers and weak expression in the basal layer [30]. Studies of Cx43 expression in the stratified epithelia of the buccal cavity demonstrate a differentiated distribution. In mice and humans, Cx43 occurs in the basal and spinous layers, and in the lingual epithelium of the hamster, it occurs in cells of the basal layer, the lower part of the squamous layer, and occasionally in the granular layer [25,28,30]. Our study indicates that expression of Cx43 in the ortho- and parakeratinized epithelium of the tongue in birds is slightly similar to that of the epithelium of the buccal cavity in the mouse.

Cx26, 30, and 43, as well as Cx31 and 40, were widely identified in the epidermis. Cx26 in mouse and rat epidermis shows weak expression only in suprabasal layers [1,6,27,29,30]. In contrast, Cx26 in the human epidermis is absent [24]. Thus, the expression profile of Cx26 in the ortho- and parakeratinized epithelium of the avian tongue is similar to that of the epidermis in the mouse and rat. Studies of Cx30 in the mouse epidermis describe its presence only in the granular layer [29]. In contrast, in the interfollicular and palm epidermis of humans, Cx30 occurs in the spinous and granular layers [26]. Significantly, Cx30 expression in the palm epidermis is stronger than in the interfollicular epidermis [26]. These results indicate that the expression profile of Cx30 in the ortho- and parakeratinized epithelium of the avian tongue is similar to that of the epidermis in humans. Importantly, the more cornified orthokeratinized epithelium shows stronger expression with Cx30 than the parakeratinized epithelium, which can be related to differences in expression between the palm and interfollicular epidermis. The analysis of Cx40 expression in mouse epidermis differs from that in avian lingual epithelia, as it indicates the absence of Cx40 or weak expression only in the basal layer of the epidermis [29,30]. In contrast, in human epidermis, Cx40 occurs in the spinous and granular layers of interfollicular and palm epidermis, which correspond to the intermediate layer of the bird lingual epithelia [24,26]. The Cx43 expression profile in the ortho- and parakeratinized epithelium of the tongue is similar to that of the mouse, rat, and human epidermis, where Cx43 is present in the basal layer and upper epithelial layers with the exception of the cornified layer [1,24,26,27,29,30]. In the case of Cx31, we reveal that the ortho- and parakeratinized epithelium shows a different expression profile than the epidermis in mice, rats, and humans, where strong expression is in the spinous and granular layers [26,27,29].

The current study indicates in detail the distribution of connexins within the keratinocyte membranes (Cx30, 31, 40, and 43), cytoplasm (mainly Cx26), or cell membrane and cytoplasm at the same time (Cx30, 31, 40, and 43). The only reports describing the localization of connexins are studies of the lingual epithelium in hamsters and the epidermis in humans and mice, where Cx26, 30, 40, and 43 are in cell membranes [6,24,25,26]. The presence of Cx26 and 43 in the keratinocyte cytoplasm was recorded only in the lingual epithelium of the hamster during wound healing [25]. The simultaneous occurrence of α-connexin Cx40 and 43 and β-connexin Cx26, 30, and 31 in the cell membranes of ortho- and parakeratinized epithelium may indicate homomeric/heterotypic or heteromeric/heterotypic gap junction formation. To date, data on such types of gap junctions in multilayered epithelia is lacking. It is only known that α-connexins Cx43 and 40 form heteromeric gap junctions in vascular smooth muscle cells [44]. The present results provide a basis for further analyses of the expression of genes encoding connexins in birds and their molecular structure in order to determine their phylogenetic origin. 

When the obtained results are related to information on the expression of connexins in pathologically altered epithelia of the mammalian oral cavity (i.e., oral squamous cell carcinoma and tongue papilloma) and epidermis (i.e., psoriasis, viral warts, or palmoplantar keratoderma), an increase in the expression of Cx26 and Cx43 is noted [25,28,46,47,48,49]. These studies simultaneously indicate that Cx26 is a marker of the hyperproliferative epidermis, responsible for intensive keratinocyte differentiation, and that the study of Cx43 expression can be used in humans as an independent biomarker of early changes associated with oral squamous cell carcinoma.

Hemichannels formed by the connexins are the ATP transporters [50,51,52,53]. A recent study in mammalian taste perception indicates that ATP as a neurotransmitter is essential for the transmission of information from type II cells to sensory nerve fibers [54]. Analyses showed the presence of RNA transcripts for Cx26, 30, 31, and 43 and indicated the presence of Cx30 and 43 in taste cells [55,56]. In birds, taste buds are mostly located in the epithelium of the palate and the bottom of the oral cavity, and only a few of them are located in the epithelium of the posterior part of the tongue, in the area of the lingual glands [57,58,59]. To date, no studies have been undertaken to determine the mechanism of taste perception in birds, so the present study indicating the presence of connexins 26, 30, 31, and 43 in the epithelia of the avian tongue provides a starting point for further studies on taste transduction in birds.

### 4.2. The Process of Cornification of the Ortho- and Parakeratinized Epithelium

Gap junctions as trans-membrane junctions composed of connexins are responsible for the transport of metabolites and ions of low molecular weight (<1 kDa), i.e., cAMP, P3, DAG, and Ca^2+^, which are involved in keratinocyte differentiation [5,6,7,8,9,10]. The calcium ions regulate the differentiation of stratified epithelia and initiate the cornification process [60,61,62]. The Ca^2+^ concentration in the intercellular space and in the cytoplasm of epidermal keratinocytes is the lowest in the basal and spinous layers, which ensures an adequate level of keratinocyte proliferation [60,61,62]. Scott et al. [4] indicated that the increase in keratinocyte proliferation is also influenced by weak expression of Cx43.

Further processes of keratinocyte differentiation and cornification require a higher calcium concentration [60,61,62]. The increase in calcium concentration in the upper layers of the epidermis is associated with the release of intracellular stores from the endoplasmic reticulum by IP3, whose molecules are transported across the gap junction and are present in the cell membrane, from where they are released into the cytosol. The present study in birds reveals that Cx26, 31, and Cx43 are weakly expressed or are absent in the basal layer of the ortho- and parakeratinized epithelium, and only Cx30 and 40 show medium-to-strong expression. Additionally, some of the connexins, e.g., Cx26 and Cx43, are present only in the cell cytoplasm, which may indicate evidence of ongoing synthesis, without gap junction formation. In the intermediate layer of both types of lingual epithelia, the expression of Cx30, 40, and 43 increases, and connexins occur in the cell membrane, where they create gap junctions. Such observations may indicate the possibility of maintaining a low Ca^2+^ ion concentration in the basal layer and a calcium gradient in both types of cornified lingual epithelia to determine the appropriate level of cell proliferation.

Generally, the epithelium of the oral cavity regenerates faster after damage than the epidermis. In contrast to the epidermis, during repair, the expression of Cx26, 30, and Cx43 is downregulated in all epithelial layers [25,29,30,63]. We identify a weak expression of Cx26 throughout the ortho- and parakeratinized epithelium of the birds’ tongues. We also reveal that the level of Cx30 expression in the parakeratinized epithelium is lower than in the orthokeratinized epithelium, which may indicate that the cells of the parakeratinized epithelium may migrate faster. This is important for a constantly desquamating parakeratinized epithelium. During the cytodifferentiation of the keratinocytes in the ortho- and parakeratinized epithelium of the avian tongue, cells in the intermediate layer change their shape [34,35,36,37,38,39]. Firstly, they become polygonal and then flatten. The arrangement of the cytoskeleton, including microtubules, is reorganized. In fibroblasts and liver epithelial cells, Cx43 binds to tubulin proteins, thereby affecting microtubule organization [64,65,66]. The current study shows the presence of Cx43 in the keratinocyte cytoplasm in the intermediate layer of both types of cornified lingual epithelia, which may be related to the reorganization of the keratinocyte cytoskeleton during the change of shape.

Cytokeratins and other proteins of the cornification process are synthesized in the intermediate layer of the cornified epithelia of the avian tongue. Skieresz-Szewczyk et al. [40,41,42] indicate the presence of avian-specific alpha- and beta-keratin, as well as KAPs (filaggrin and loricrin) and TGM-1 in the intermediate layer of both types of cornified lingual epithelia. The presence of kinase C is required to initiate the synthesis of the above proteins. Kinase C is activated by DAG and attaches calcium ions, which begins the synthesis. As mentioned earlier, DAG can be transported via the gap junction.

The present study identifies that in the lower part of the intermediate layer, Cx43 in the parakeratinized epithelium and Cx30, 31, and 40 in both types of the lingual epithelia are present in the cell membrane. This fact indicates the formation of two functional hemichannels, ensuring adequate communication between the keratinocytes and, thus, synchronization of the cornification process. Cx30 and 40 in the upper part of the intermediate layer in both types of cornified epithelia, and Cx43 in the orthokeratinized epithelium, occur both in the cell membrane and cytoplasm. This distribution may indicate the simultaneous presence of functional gap junctions and the presence of connexins in the cytoplasm, which may indicate progressive degradation of connexons. During the degradation of connexons, a part or all of the double-membrane channel plaque invaginates into the cell cytoplasm and forms sub-membrane intracellular annular junctions, which are then destroyed by lysosomes or proteasomes [5,11]. Interestingly, the expression of Cx31, 40, and 43 increases in the upper part of the intermediate layer of the orthokeratinized epithelium and decreases in the parakeratinized epithelium. It may indicate that communication between keratinocytes and the cornification process in the orthokeratinized epithelium is continued, while in the parakeratinized epithelium, it is gradually reduced.

The cornified layer, which is the final product of the cornification process of the multilayered epithelia, in the orthokeratinized epithelium is built up by strongly flattened keratinocytes devoid of cell nuclei and organelles [34,35,36,37,38,39]. The superficial cells of the orthokeratinized epithelium exfoliate as scales. In contrast, the cornified layer of the parakeratinized epithelium is made up of flattened keratinocytes with degenerating cell nuclei and single organelles, and the superficial cells exfoliate as continuous plates [34,35,36,37,38,39]. The present study shows that connexins in the cornified layer of the orthokeratinized epithelium are generally absent, and only in some keratinocytes in the lower part of the cornified layer do they demonstrate the presence of Cx30 and 31 in the cell cytoplasm, which may indicate gap junction degradation. In the parakeratinized epithelium, Cx40, Cx30, and 31 occur both in the cell cytoplasm and in the cell membrane, which may reveal the presence of single functional gap junctions and the maintenance of continuous integrity between the keratinocytes of the cornified layer.

## 5. Conclusions

The IHC analyses of the distribution of connexins in the ortho- and parakeratinized epithelium of the avian tongue reveal the presence of α-connexins Cx40 and 43 and β-connexins Cx26, 30, and 31 characteristics of the mammalian epidermis and multilayered epithelia of the oral cavity, which may explain the ectodermal origin of these epithelia. The low expression of the studied connexins in the basal layer and their increase in the upper layers of both types of epithelia may determine the suitable conditions for cell proliferation and renewal of the multilayered epithelia. The α-connexins Cx40 and 43 and β-connexin Cx30 are involved in the initiation and synchronization of the cornification process in studied cornified epithelia. The orthokeratinized epithelium shows stronger expression of Cx31, 40, and 43 in the upper part of the intermediate layer than the parakeratinized epithelium, which may suggest continuous communication of keratinocytes in relation to progressive cornification, resulting in the formation of a thick cornified layer. The cornified layer generally showed an absence of the studied connexins, which may indicate a loss of communication between cells before their peeling. In turn, expression of connexins in keratinocytes of the superficial layer in parakeratinized epithelium is a sign of the maintenance of cell-to-cell communication. The presented studies, particularly the data on the expression of Cx26 and Cx43, may be relevant, as in mammals, in the comparative study of the normal and pathological conditions of the oral cavity epithelia in birds.

## Figures and Tables

**Table 1:** Distribution of the β-connexins Cx26, 30, and 31 in the ortho- and parakeratinized epithelium in the duck, goose, and domestic turkey. +++ strong color reaction, ++ medium color reaction, + weak color reaction, - lack of color reaction, −/+ lack or weak color reaction; cc—cell cytoplasm; cm—cell membrane.

Cx26								
Species	Basal Layer		Intermediate Layer -Lower Part		Intermediate Layer -Upper Part		Cornified Layer	
	Ortho-	Para-	Ortho-	Para-	Ortho-	Para-	Ortho-	Para-
Duck	-	++ cc, cm	+ cc	++ cc, cm	+ cc	+ cc, cm	-	-
Goose	-	-	+ cc	+ cc, cm	−/+ cc	+ cc, cm	-	-
Turkey	+ cc	+ cc	+ cc	+ cc, cm	+ cc	+ cc, cm	−/+ cm	-
**Cx30**								
**Species**	**Basal Layer**		**Intermediate Layer** **-Lower Part**		**Intermediate Layer** **-Upper Part**		**Cornified Layer**	
	**Ortho-**	**Para-**	**Ortho-**	**Para-**	**Ortho-**	**Para-**	**Ortho-**	**Para-**
Duck	++ cm	++ cm	+++ cm	+/++ cm	+++ cc, cm	+ cc, cm	−/+ cc	+ cc, cm
Goose	++ cm	++ cm	+++ cm	+/++ cm	++/+++ cc, cm	+ cc, cm	−/+ cc	+ cc, cm
Turkey	-	+ cc	++ cm	++ cm	++ cc, cm	+ cc, cm	−/+ cc	++ cc
**Cx31**								
**Species**	**Basal Layer**		**Intermediate Layer** **-Lower Part**		**Intermediate Layer** **-Upper Part**		**Cornified Layer**	
	**Ortho-**	**Para-**	**Ortho-**	**Para-**	**Ortho-**	**Para-**	**Ortho-**	**Para-**
Duck	+ cm	+ cm	+ cm	+ cm	++ cc, cm	+ cc, cm	−/+ cc	+ cc, cm
Goose	++ cm	+ cm	+ cm	+ cm	++ cc, cm	+ cc, cm	−/+ cc	-
Turkey	+ cm	-	+ cm	−/+ cc, cm	++ cc, cm	−/+ cc, cm	−/+ cc	++ cm

**Table 2:** Distribution of the α-connexins Cx40 and 43 in the ortho- and parakeratinized epithelium in the duck, goose, and domestic turkey. +++ strong color reaction, ++ medium color reaction, + weak color reaction, - lack of color reaction, −/+ lack or weak color reaction, cc—cell cytoplasm, cm—cell membrane.

Cx40								
Species	Basal Layer		Intermediate Layer -Lower Part		Intermediate Layer -Upper Part		Cornified Layer	
	Ortho-	Para-	Ortho-	Para-	Ortho-	Para-	Ortho-	Para-
Duck	+ cm	+++ cm	+ cm	+++ cm	++ cc, cm	+ cm	-	+ cc
Goose	+ cm	+++ cm	++ cc, cm	+++ cm	++ cc, cm	+ cc, cm	-	−/+ cc, cm
Turkey	+ cm	++ cm	+ cm	++ cm	++ cc, cm	+ cc, cm	-	+ cc, cm
**Cx43**								
**Species**	**Basal Layer**		**Intermediate Layer** **-Lower Part**		**Intermediate Layer** **-Upper Part**		**Cornified Layer**	
	**Ortho-**	**Para-**	**Ortho-**	**Para-**	**Ortho-**	**Para-**	**Ortho-**	**Para-**
Duck	+ cc	+ cm	+ cc, cm	+++ cm	+++ cc, cm	−/+ cm	-	-
Goose	+ cc	+++ cm	+ cc, cm	+++ cm	+++ cc, cm	−/+ cm	-	-
Turkey	-	+ cm	++ cc, cm	++ cm	+++ cc, cm	−/+ cc, cm	-	+ cc, cm

## Data Availability

The data presented in this study are available upon request from the corresponding authors. The data are not publicly available due to privacy or ethical restrictions.

## Supplementary Materials

The following supporting information can be downloaded at: https://www.mdpi.com/article/10.3390/cells12131776/s1, Figure S1: The cross-section of (a–d) the orthokeratinized epithelium and (e–g) the parakeratinized epithelium. IHC staining of the Cx26. Bl—basal layer; Lp—lamina propria of mucosa; LInt—the lower part of the intermediate layer; UInt—upper part of the intermediate layer; Cl—cornified layer. Arrows point to the positive color reaction between connective tissue papillae (asterisks). Figure S2: The cross-section of (a–d) the orthokeratinized epithelium and (e–g) of the parakeratinized epithelium. IHC staining of the Cx30. Bl—basal layer; Lp—lamina propria of mucosa; LInt—the lower part of the intermediate layer; UInt—upper part of the intermediate layer; Cl—cornified layer. Arrows indicate the positive color reaction between connective tissue papillae (asterisks). Figure S3: The cross-section of (a–d) the orthokeratinized epithelium and (e–g) the parakeratinized epithelium. IHC staining of the Cx31. Bl—basal layer; Lp—lamina propria of mucosa; LInt—the lower part of the intermediate layer; UInt—upper part of the intermediate layer; Cl—cornified layer. Arrows point to the positive color reaction between connective tissue papillae (black asterisks). Red asterisk marks the area where only part of the cells reveal a positive color reaction. Figure S4: The cross-section of (a–d) the orthokeratinized epithelium and (e–g) the parakeratinized epithelium. IHC staining of the Cx40. Bl—basal layer; Lp—lamina propria of mucosa; LInt—the lower part of the intermediate layer; UInt—upper part of the intermediate layer; Cl—cornified layer. Arrows indicate the positive color reaction between connective tissue papillae (asterisks). Figure S5: The cross-section of (a–d) the orthokeratinized epithelium and (e–g) the parakeratinized epithelium. IHC staining of the Cx43. Bl—basal layer; Lp—lamina propria of mucosa; LInt—the lower part of the intermediate layer; UInt—upper part of the intermediate layer; Cl—cornified layer. Arrows point to positive color reaction between connective tissue papillae (asterisks).
