# Supplementary material for: Pattern Distribution of Connexins in the Ortho- and Parakeratinized Epithelium of the Lingual Mucosa in Birds
Source: Cells. 2023 Jul 4;12(13):1776. doi: 10.3390/cells12131776 (PMC10341081; doi:10.3390/cells12131776)
Supplement: Supplementary file 1 [file cells-12-01776-s001.zip › cells-2415824-supplementary.pdf]

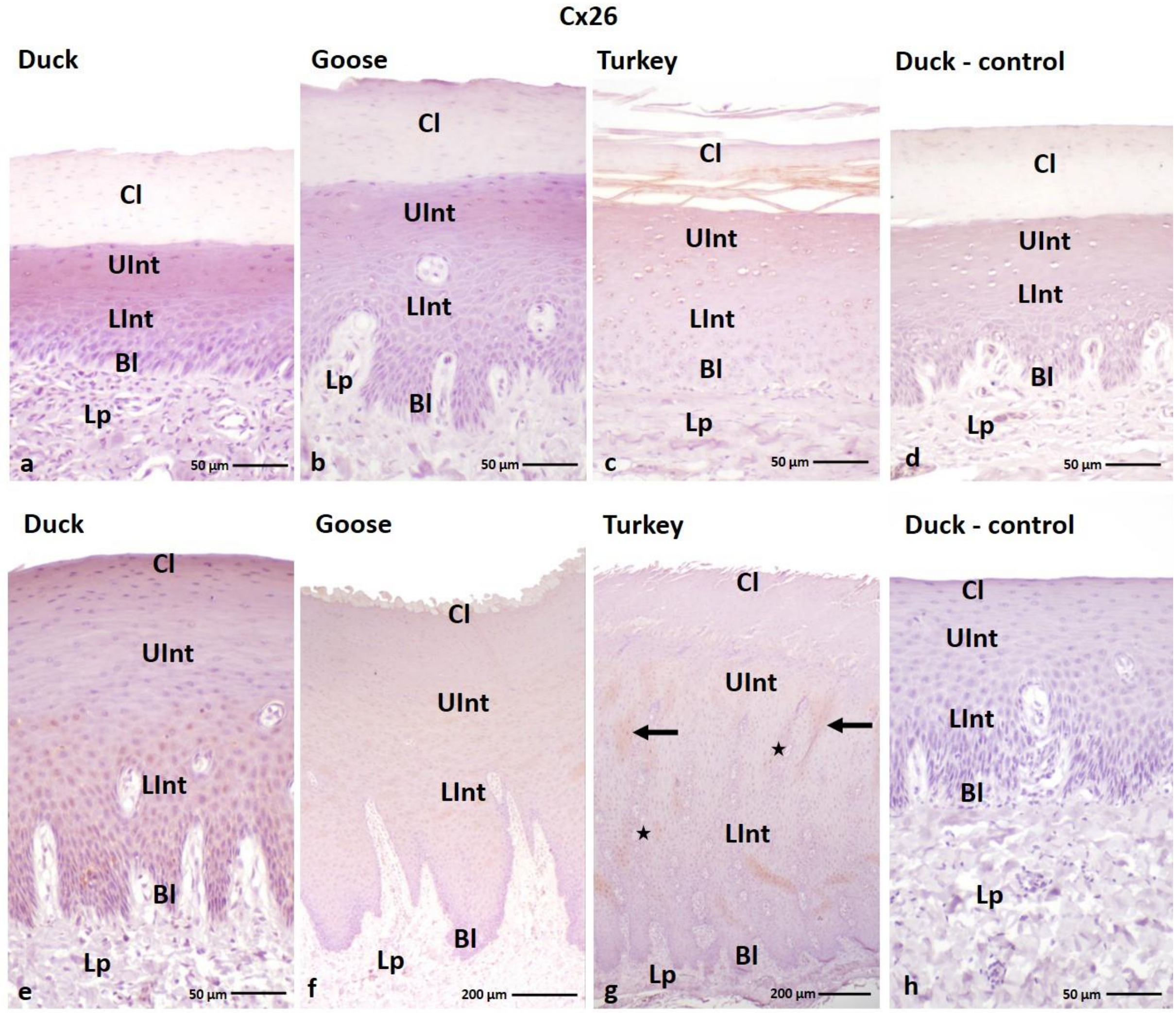

Figure S1. The cross-section of (**a** - **d**) the orthokeratinized epithelium and (**e** - **h**) the parakeratinized epithelium. IHC staining of the Cx26. Bl - basal layer, Lp - lamina propria of the mucosa, LInt - the lower part of the intermediate layer, UInt - upper part of the intermediate layer, Cl - cornified layer. Arrows point to the positive colour reaction between connective tissue papillae (asterisks).

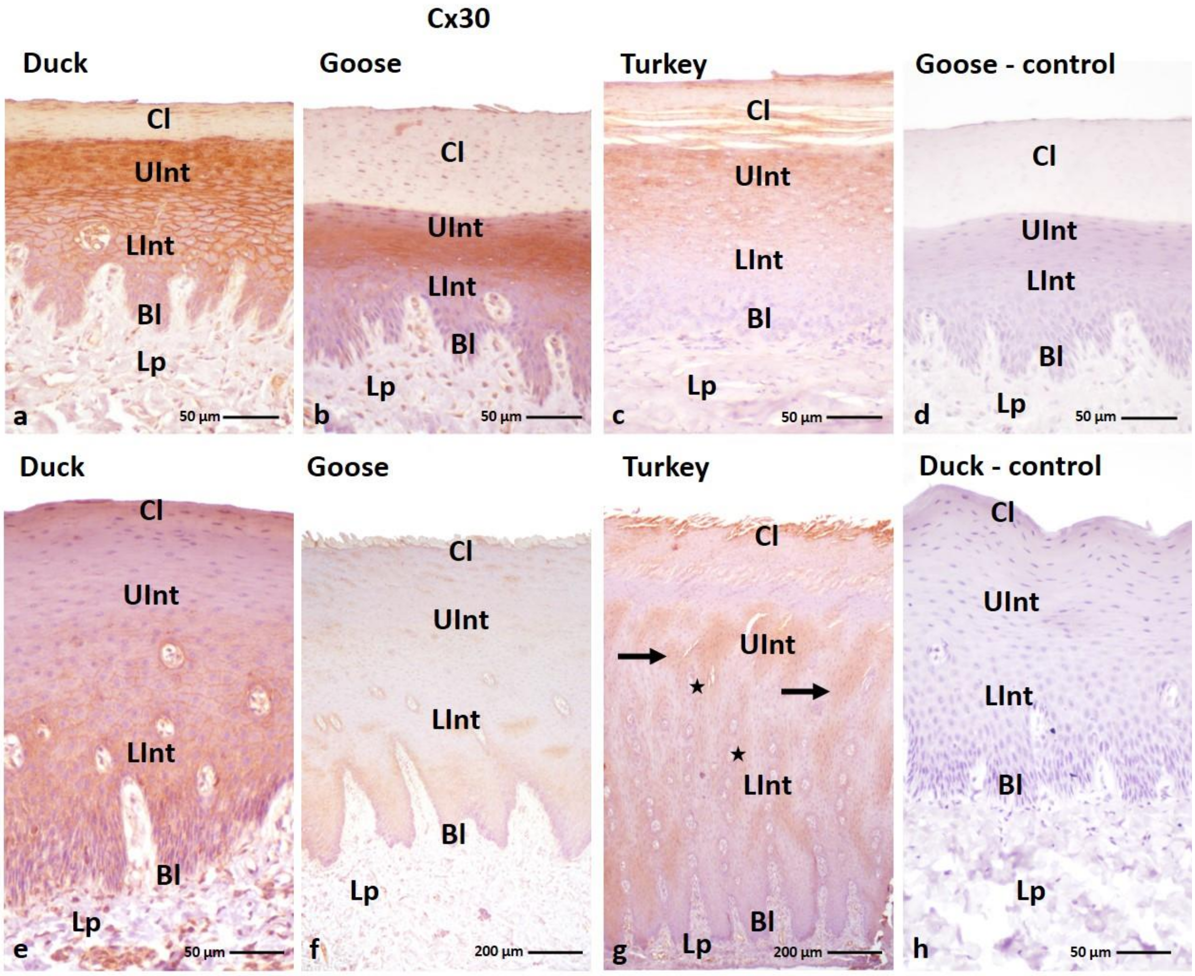

Figure S2: The cross-section of (**a - d**) the orthokeratinized epithelium and (**e - h**) of the parakeratinized epithelium. IHC staining of the Cx30. Bl - basal layer, Lp - lamina propria of the mucosa, LInt - the lower part of the intermediate layer, UInt - upper part of the intermediate layer, Cl - cornified layer. Arrows indicate the positive colour reaction between connective tissue papillae (asterisks).

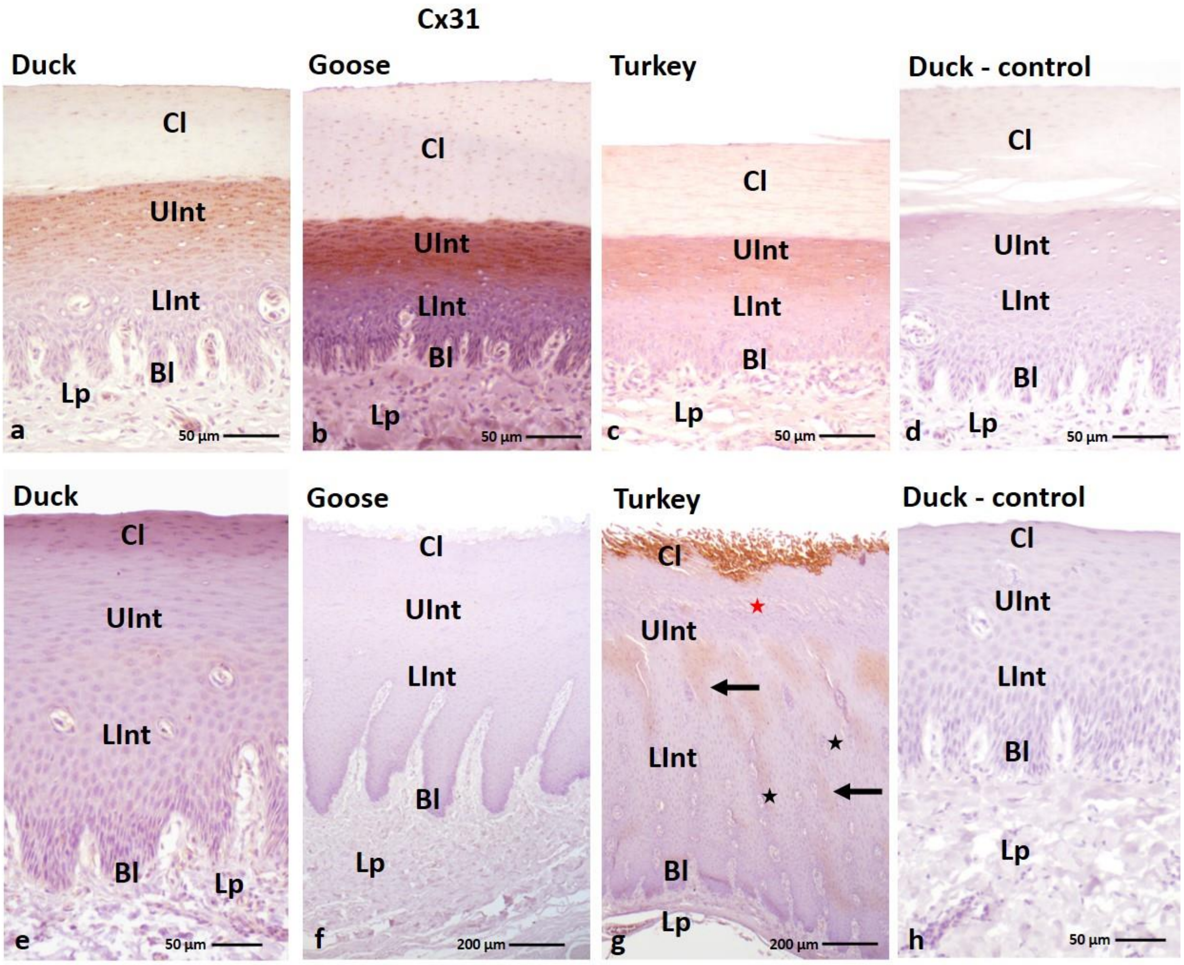

Figure S3: The cross-section of (**a - d**) the orthokeratinized epithelium and (**e - h**) the parakeratinized epithelium. IHC staining of the Cx31. Bl - basal layer, Lp - lamina propria of the mucosa, LInt - the lower part of the intermediate layer, UInt - upper part of the intermediate layer, Cl - cornified layer. Arrows point to the connective tissue papillae. Arrows point to the connective tissue papillae. Arrows point to the positive colour reaction between connective tissue papillae (black asterisks). Red asterisk marks the area where only part of the cells reveal a positive colour reaction.

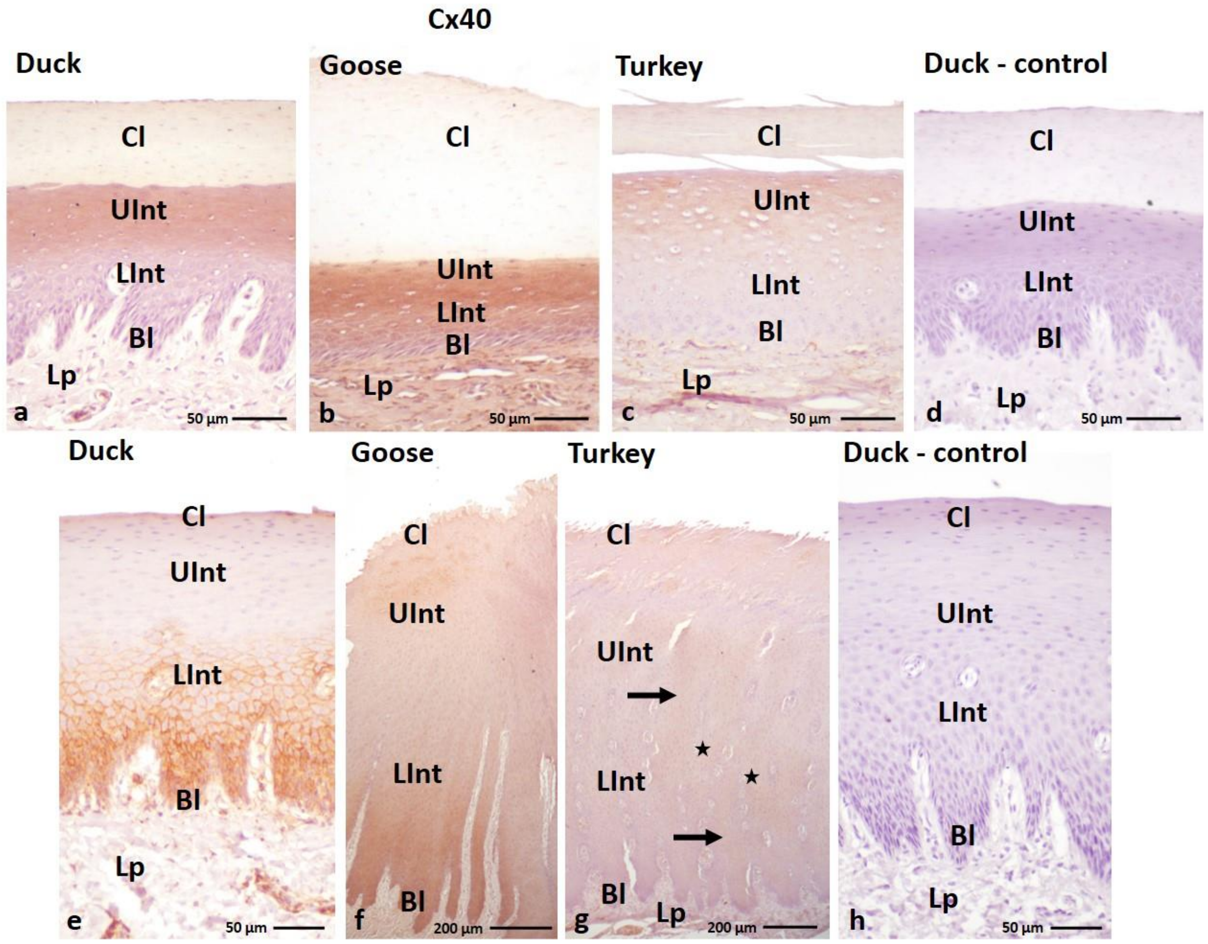

Figure S4: The cross-section of (**a** - **d**) the orthokeratinized epithelium and (**e** - **h**) the parakeratinized epithelium. IHC staining of the Cx40. Bl - basal layer, Lp - lamina propria of the mucosa, LInt - the lower part of the intermediate layer, UInt - upper part of the intermediate layer, Cl - cornified layer. Arrows indicate the positive colour reaction between connective tissue papillae (asterisks).

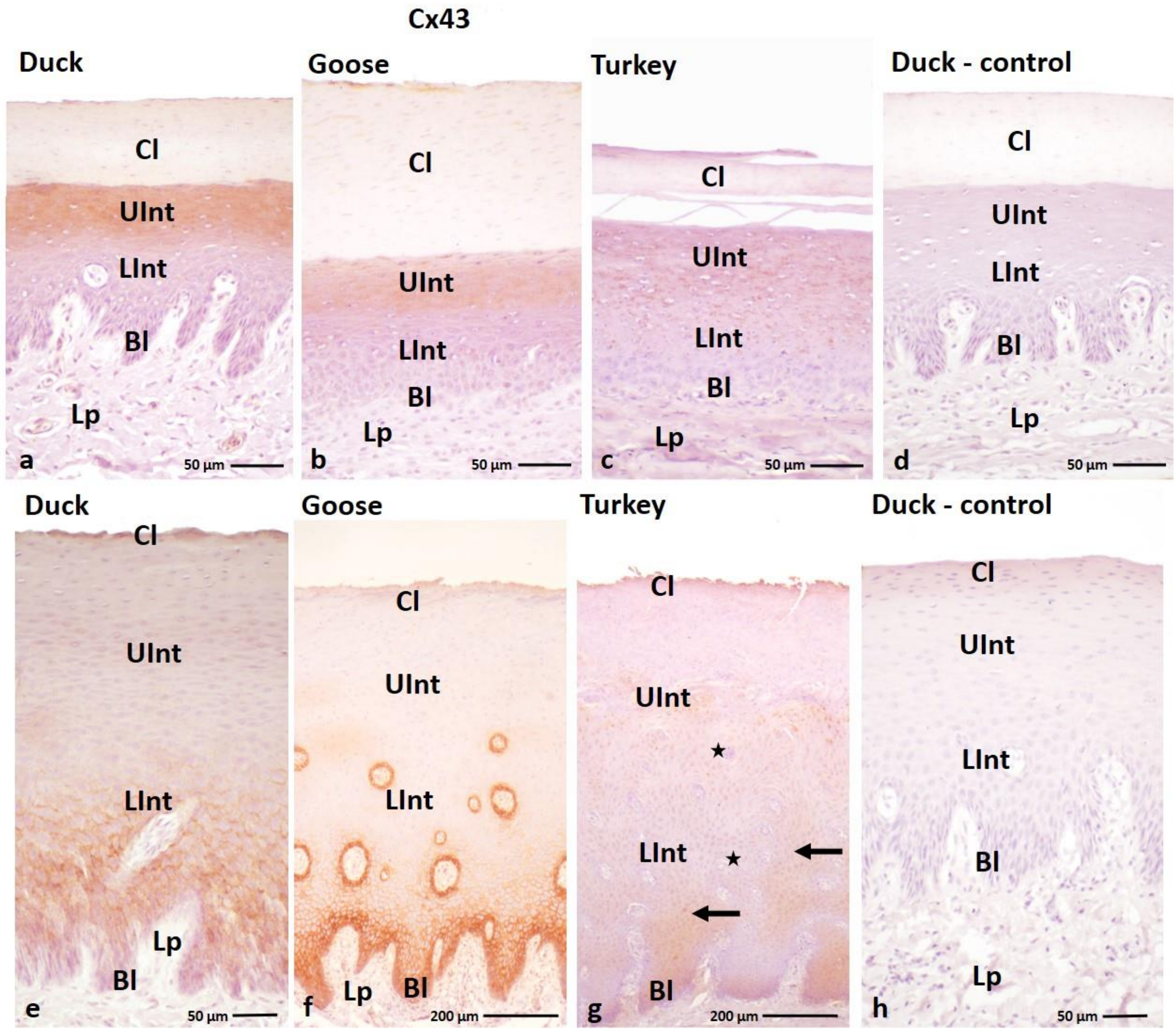

Figure S5: The cross-section of **a - d**) the orthokeratinized epithelium and **e - h**) the parakeratinized epithelium. IHC staining of the Cx43. Bl - basal layer, Lp - lamina propria of the mucosa, LInt - the lower part of the intermediate layer, UInt - upper part of the intermediate layer, Cl - cornified layer. Arrows point to positive colour reaction between connective tissue papillae (asterisks).
